# Family physicians’ professional identity formation: a study protocol to explore impression management processes in institutional academic contexts

**DOI:** 10.1186/1472-6920-14-184

**Published:** 2014-09-06

**Authors:** Charo Rodríguez, Teresa Pawlikowska, Francois-Xavier Schweyer, Sofia López-Roig, Emmanuelle Bélanger, Jane Burns, Sandrine Hugé, Maria Ángeles Pastor-Mira, Pierre-Paul Tellier, Sarah Spencer, Laure Fiquet, Inmaculada Pereiró-Berenguer

**Affiliations:** Department of Family Medicine, Faculty of Medicine, McGill University, Montreal, Quebec Canada; Health Professions Education Centre, Royal College of Surgeons in Ireland, Dublin, Ireland; École des Hautes Études en Santé Publique, School of Public Health, University of Rennes 1, Rennes, France; Équipe de Recherche sur les Inégalités de Santé, Centre Maurice Halbwachs (UMR 8097 CNRS-EHESS-ENS), Paris, France; Department of Health Psychology, Miguel Hernández University, Elche, Alicante Spain; Department of General Medicine, University of Rennes 1, Rennes, France; The London School of General Practice, Queen Mary University of London, London, UK; Puerto de Sagunto Community Health Center, Puerto de Sagunto, Valencia, Spain

**Keywords:** Family physicians, Professional identity formation, Institutional theory, Impression management, Case study, Rhetorical analysis

## Abstract

**Background:**

Despite significant differences in terms of medical training and health care context, the phenomenon of medical students’ declining interest in family medicine has been well documented in North America and in many other developed countries as well. As part of a research program on family physicians’ professional identity formation initiated in 2007, the purpose of the present investigation is to examine in-depth how family physicians construct their professional image in academic contexts; in other words, this study will allow us to identify and understand the processes whereby family physicians with an academic appointment seek to control the ideas others form about them as a professional group, i.e. impression management.

**Methods/Design:**

The methodology consists of a multiple case study embedded in the perspective of institutional theory. Four international cases from Canada, France, Ireland and Spain will be conducted; the "case" is the medical school. Four levels of analysis will be considered: individual family physicians, interpersonal relationships, family physician professional group, and organization (medical school). Individual interviews and focus groups with academic family physicians will constitute the main technique for data generation, which will be complemented with a variety of documentary sources. Discourse techniques, more particularly rhetorical analysis, will be used to analyze the data gathered. Within- and cross-case analysis will then be performed.

**Discussion:**

This empirical study is strongly grounded in theory and will contribute to the scant body of literature on family physicians’ professional identity formation processes in medical schools. Findings will potentially have important implications for the practice of family medicine, medical education and health and educational policies.

**Electronic supplementary material:**

The online version of this article (doi:10.1186/1472-6920-14-184) contains supplementary material, which is available to authorized users.

## Background

The purpose of the present investigation, granted by the Canadian Institutes of Health Research (CIHR MOP-125906) is to examine in-depth how family physicians construct their professional *image* in academic medical contexts; in other words, the processes whereby family physicians seek to control the ideas others form of them as a professional group, i.e. impression management [1, 2]. The project makes up part of an international research program on family physicians’ professional *identity* initiated with a 2007 CIHR-funded investigation (MOP-85044) in which we studied processes of medical student *identification* with family medicine in medical schools [3–5]. The trend that called for us to spearhead such research program was medical students’ declining interest in family medicine as a career choice.

This disturbing trend is also demonstrated by the health comparative statistics from the 30 countries of the Organization for Economic Co-operation and Development (OECD). The number of medical specialists rose by 60% between 1990 and 2007, while the number of general practitioners increased only by 23% [6]. So far, Canada has been able to maintain a balance, i.e. about 50% each [7]. However, the equilibrium between specialists and generalist in the country has been seriously threatened since 1992, when the proportion of Canadian graduates who chose family medicine as 1^st^ choice in the residency match started decreasing to attain a minimum of 24.8% in 2003 [8]. More recently this negative trend has reversed, reaching 31.8% in 2010 [9] and 34.0% in 2011 (1^st^ iteration) [10]. Yet, while encouraging, these percentages are far from the national goal of 50% [11]. Furthermore, the number of vacant positions in family medicine residency programs still remains very high: in 2013, 40% of vacancies at the 1^st^ iteration were in family medicine [10].

As noted above, and despite significant differences in terms of medical training and health care context, the phenomenon of medical students’ declining interest in family medicine has also been documented in many other developed countries such as the United States [12, 13], Australia [14], the United Kingdom [15], France [16], Switzerland [17], and Spain [18]. A gap between the income of specialists and family physicians, advances in specialized medical technology, and a loss of professional prestige [19–25] have been documented as factors than can help explain this trend.

However, the influence of institutional discourses on the construction and reconstruction of family physicians’ professional identity in academic centres during undergraduate medical training and its influence on career choice has been largely ignored. Our international research program was conceived to fulfil this research gap. In the first aforementioned investigation, we provided sound evidence about the relationship between *professional reputation*, discursively constructed, and students’ *identification* with family medicine practice. More specifically, the study highlighted the clear polarization existing between the medical school in which family medicine was a valued academic discipline, to which students were exposed from the very beginning of their studies (the British case), and those in which students had little or no exposure to this practice and where family medicine was disregarded as a valid career option (Canadian, French and Spanish cases). In the former, the reputation of the profession was very high, the features of this professional practice as well as the knowledge and skills necessary to perform as a GP appearing to be held in high esteem by both students and educators. In this institutional context, the majority of students identified with general practice and chose this medical field as career choice. It was the opposite in the other three medical schools, where family medicine was lessened, either overtly or through a double academic discourse that stressed the importance of the practice for the health care system while denigrating family medicine because of its lack of a hard medical skill set [3].

Nevertheless "[r]eputations rise and fall" [26]. Put differently, as identity, reputation and image are intimately related processes [27–29], family physicians may be extremely motivated to project on others an enhanced perception of what they are as a medical professional group (image) with the aim to improve how the ideas the others have about them as a professional group (reputation) as well as their own sense of self (identity) and the future identification of physicians-to-be with the profession; those are the processes that, within institutional academic contexts, our international research team proposes to focus on in the present investigation.

### Literature review

#### Identity and image

The Oxford Dictionary defines ‘identity’ as the "condition of being a specified person or thing". As a topic of investigation, identity has been very popular among organizational scholars. In this field of inquiry, identity has effectively been treated from many different perspectives, e.g. social identity theory; embedded inter-group theory; race, ethnicity and gender research; organizational demography; ethnology [30]. In recent times, there is a renewed concern by identity issues, largely justified by current broader changes in society (globalization, the expansion of information technology, and greater diversity), the decline of bureaucratic organizational forms, and a special interest in power and meaning.

In 1985 Albert and Whetten published a landmark article that has strongly influenced the study of identity [31]. Being among the first to define the term as a "clear, distinctive, important, useful and measurable" construct, the authors provoked such a noteworthy impact that most later treatments of the topic were built on their work [28]. In effect, an extensive stream of work on identity has viewed it as a stable and well-defined element. Although this traditional view of identity is still espoused, recent perspectives tend to see identity as a social process in constant reconstruction [30]. Identity would therefore not be stable or fixed but socially and historically constructed and subject to contradictions, revisions, and change. This new approach to identity also implies that the focus of interest is more on the social aspects of the self than on the individuated self-concept of identity (see the Oxford Dictionary’s definition above), the latter being adopted by most social psychological theories of the self in the past [32]. Identity is therefore conceptualized as an ongoing process that encompasses the ‘sense of self’ created through social interactions. In other words, identity concerns how social actors understand and explain themselves through dynamic interactional processes [30, 33, 34]. In addition, the accomplishment of these interactional processes is mainly made through language-in-use. In other words, actors’ discourses are constitutive of their own identity and of the space and role they occupy in the social world [35]. As Ainsworth notes [36]: "Discourse constructs social identity by defining groups, groups’ interests, their position within society, and their relationship to other groups" (p. 31).

If one accepts the assumption that identity is constructed through social interactions, then two other levels of representation of the self besides the individual level can be identified, namely interpersonal and group levels [32]. At the interpersonal level, the relational self is typically constructed through dyadic relationships with significant others; at the group level, the collective self, which does not require personal relationships among the group members, involves the sense of belonging to a particular social category. As noted by Brewer and Gardner [32], some social identities can be constructed either through interpersonal relationships or collective identities. This is the case for the medical professional identity, which can be built through the doctor-patient relationship, but also in terms of membership in the social category of the medical profession.

The relational nature of identity intimately connects this concept with the notion of ‘image’. For Dutton and Dukerich [37] image is what organizational members believe other think about their organization. According to Hatch and Schultz [38], marketing literature has put forward a more external definition of image, considering it as the views of the organization held by various external constituencies such as the customers, suppliers and regulators. Indeed, a comprehensive definition of image that encompasses both perspectives is provided by Alvesson [39], for him image is the bright impression held by an actor towards an organization that has been nourished by the organization’s projected portrait of itself (see also Hatch and Schultz [38]). One could therefore assert that identity and image are different but interpenetrated organizational dimensions, which emerge from the conversational process between internal and external organizational stakeholders [28, 38].

#### Professional identity and professional image

Sociologists and organization scholars seem to be more and more interested in exploring the crossroad of professions and organizations (see for example the recent monograph of *Current Sociology* on this topic, introduced by its editors, Muzio and Kirkpatrick [40] entitled ‘Professions and organizations – a conceptual framework’). A ‘new professionalism’ is even advocated as professionals are working increasingly in large-scale workplaces, yet international firms [41]. Still professionals are for some "the preeminent institutional agents of our time" [42]. A ‘profession’ is an occupation characterized by both the possession of a specialized body of knowledge and a commitment to service [43–45]. As any other collective self, the medical profession: "reflects internalizations of the norms and characteristics of important reference groups and consists of cognitions about the self that are consistent with that group identification" [32]. This process of differentiation, which comes into being through language-in-use, implies attaching value to a particular group membership [46] and simultaneously a separation from other social groups that are not only "different" but usually also "less valuable" [47]. A professional identity is therefore created and recreated through professional discourse. According to Sarangi and Roberts [43], professional discourse would be constituted by everything professionals do in the day-to-day accomplishment of their responsibilities and tasks. Such a discourse would be "not only durable, but also legitimate and authoritative" (p. 15). It is also important to note that professional discourses do not emerge in a vacuum. On the contrary, the fleshing out of professional identity through discursive activity can only be understood and explained within the context in which social interactions are performed. In this regard, we agree with Sarangi and Roberts [43] when they point out: "What counts as legitimate professional discourse will depend on the range of discourses available within an institutional order" (p. 15). Context is thus crucial for both understanding discourses and for creating identity [34].

It is important to note that professional identity construction starts in educational institutions such as medical schools where trainees internalize the norms, values and power relations that characterize the collective identity of the profession to which they aspire to be part of. There, a professional trainee learns and assumes the particular discourse of the profession of his/her choice in order to become a legitimated and credible member of that profession. Scholars have conceptually and empirically examined the ideological socialization of medical students in academic centers for more than 40 years [48–54]. However, as recently noted by Cooke and collaborators [55] with regard to medical training in the US, professional identity formation is one of the most important, yet most neglected strategy for reforming medical school and residency.

Professional identity and professional image are also two interrelated constructs. In effect, there is no doubt that what professionals identify about their professional group is influenced by the way others see them. Concomitantly, professionals can project a particular perception of what they are as a professional group. Grounded in self-reflecting appraisals, professional image refers to "an externally oriented, public persona" and is defined as "the aggregate of key constituents […] and perceptions of one’s competence and character" [56]. Indeed, professional image has important consequences in terms of social reward and career success [1, 57, 58] since, as noted by Roberts [56], "people who construct viable professional images are perceived as being capable of meeting the technological and social demands of their jobs" (p. 687). Processes of professional image construction therefore appear crucial to reach and preserve social reputation and legitimacy.

#### Impression management

Impression management refers to the processes by which social actors try to create, maintain or modify the ideas others have about them [1–60]. The roots of this concept can be found in Goffman’s notion of self-presentation [61]. According to this sociologist, people usually seek information about others in their social space. The sources of this information can be different, one of them being the individual him/herself (self-presentation). That is, the individual will wittingly or unwittingly express him/herself to others, and the others will have to be impressed somehow by him/her. Using the metaphor of the play, for Goffman self-presentation involves an actor and its audience interacting in a particular context and jointly defining a particular situation, with the actor selecting the behavior that s/he expects will generate the best impression in the audience.

In the 1960’s, social psychologists were also attracted by the conceptualization and empirical research on impression management [62–65]. In this corpus of texts, the frequently-cited work by Leary and Kowalski [1] deserves to be examined in more detail. Being interested in identifying factors that affect self-presentations to others, these authors recognize two discrete processes involved in what has been called impression management, namely impression motivation and impression construction. Among primary self-presentational motives, they consider that individuals may be motivated to control how others perceive them when they want to maximize their reward-cost ratio in social interactions, when they highly value the goals to achieve, and when there is a discrepancy between desired and current image. Then, once individuals are motivated to act, five factors might have an influence on the way they will behave in order to impress others: (1) their sense of self (i.e. identity), (2) what they desire to be or not to be, (3) social constraints with regard to the role to be played, (4) value attached to the model to be emulated, and (5) current reputation and image. This conceptual framework of impression management, which concerns cognitive/behavioral intra/interpersonal levels, emphasizes that identity, image and reputation are intertwined.

The interest in impression management among organizational scholars was scant until the 1980’s [59]. The willingness to examine this topic has increased since then, but remains mostly conceptual [66, 67], as empirical research [68–70] is still scarce. Likewise, most works have focused on a micro- individual level of analysis [71–73], the macro-organizational level [74, 75] has been considered to a lesser extent [60].

The taxonomy of organizational impression management *tactics* proposed by Mohamed and collaborators [67] has made a significant theoretical contribution in this field. Presented in a 2×2 matrice, and inspired by prior work on impression management tactics at the micro-individual level, these scholars argue that organizations may use direct/indirect and assertive/defensive tactics to construct/preserve desirable images among target audiences. What is more, organizations can use more than one tactic at a time as they are not mutually exclusive. Direct and assertive tactics (e.g., ingratiation, intimidation) are adopted when the organization actively works to create and enhance a desirable organizational image, while direct and defensive tactics (e.g. disclaimers, apologies) aim at protecting the organizational image. On the other hand, indirect impression management tactics, either assertive (e.g. boasting) or defensive (e.g. blurring), seek to manage information about the people and things the organization is associated with.

More recently, Robert [56] has interestingly theorized about impression management for constructing *professional image* in organizational settings. Drawing on social identity, impression management and organizational behavior theories, this author proposes a comprehensive model that encompasses both individual and group levels and includes three main components: (1) impression monitoring, i.e. the awareness of how one is perceived in a given situation is generated; (2) impression motivation, i.e. how identity threats and negative image discrepancies trigger actors’ desire to adopt impression management; and (3) impression construction, i.e. how actors effectively enact their personal and social identities in order to create their desired professional images. Furthermore, this framework also highlights intended and unintended consequences of impression management behaviors at the individual, interpersonal, group and organizational performance levels.

#### Summary of the literature review

In our contemporary globalized world, individual, group and organizational identity are constructs that have attracted increasing attention among scholars from different disciplines. In organizational and management literature, current trends conceive identity and related concepts, i.e. image and reputation, as constructed through social interactions, mostly of a discursive nature. Professionals are leading social actors embedded in organizational fields; therefore the construction of professional identities appears of particular interest not only for themselves as cohesive groups but also for the institutions in which they operate. Two important issues are at stake here. First, professional identity formation are processes that begin in educational institutions (such as medical schools), where trainees espouse the norms, values and power relations akin the profession they aim to integrate. Second, the way professionals understand themselves comes basically into being through the discursive activity developed among group members and between them and external stakeholders in their situated contexts. Processes of enhanced professional image construction, i.e. impression management, do therefore allow the constant reconstruction of professional identities as well as the attainment and preservation of social reputation and legitimacy. Despite its unquestionable interest, our literature review has revealed that empirical works in organizational image construction are still scant; and there is even less research on professional image construction. To our knowledge, no study about processes of family physician professional image construction in medical schools has hitherto been conducted.

### Research questions

As noted above, the present study builds on our previous investigation in which we studied processes of medical student identification with and the reputation of family medicine in medical schools. Findings unveiled both low interest in a family medicine career pathway among medical students trained in medical schools where this profession was devaluated, and high interest among those students trained in the medical school in which general medical practice had a greater legitimacy. As part of an on-going research program on family physicians’ professional identity, we are at the present interested in examining how family physicians construct their professional image in the same academic contexts. This being said, it is important to point out that this study will be concomitant with important curricular and field-level institutional changes in all the cases involved in the investigation. Three interrelated research questions will guide the study: (1) *Why are academic family physicians motivated to control/improve their professional image in academic contexts?* Here, we are interested in describing and understanding the motivations underlying processes of family physicians professional image construction. (2) *What are the impression management strategies they have undertaken/will undertake in order to improve the image of the family medicine discipline?* The answer of this question will imply the description and comprehension of the different ways academic family physicians behave in order to enhance their professional image. (3) *How are these strategies being implemented, and what have been their intended and unintended consequences to date?* Indeed, we want to understand and explain processes of impression management adoption, as well as the consequences (projected as well as unintentional) of their adoption at the individual, interpersonal group and organizational levels.

### Theoretical framework

Taking into account identity and impression management organizational literature, *institutional theory* will be the overarching theoretical perspective we will adopt in this investigation to better describe, understand and explain processes of professional image construction occurring within given institutional contexts. We will specifically retrieve up to date literature on institutionalism that calls attention to institutional entrepreneurship, change, and power/knowledge dynamics. Institutional theory is one of the most prominent approaches for understanding organizational phenomena. What has been called neo-institutionalism traces its origins in the seminal works of Meyer and Rowan [76], Meyer and Scott [77], Tolbert and Zucker [78], Zucker [79], and Powell and DiMaggio [80, 81]. The essential characteristics of institutional theory set up by these scholars can be summarized as follows [81, 82]: all organizations undergo influences from their institutional (rationalized myths of appropriate conduct) and network/field contexts, although not all to the same extent. Isomorphism (coercive, normative or mimetic) would involve processes by which organizations try to conform to institutional pressures in order to gain legitimacy. In a particular context, a practice is institutionalized when it is widely accepted, followed, and enduring. Institutionalization is thus defined as the process by which something acquires the "rule-like status" [76], that is, it becomes taken-for-granted.

**Figure 1:**
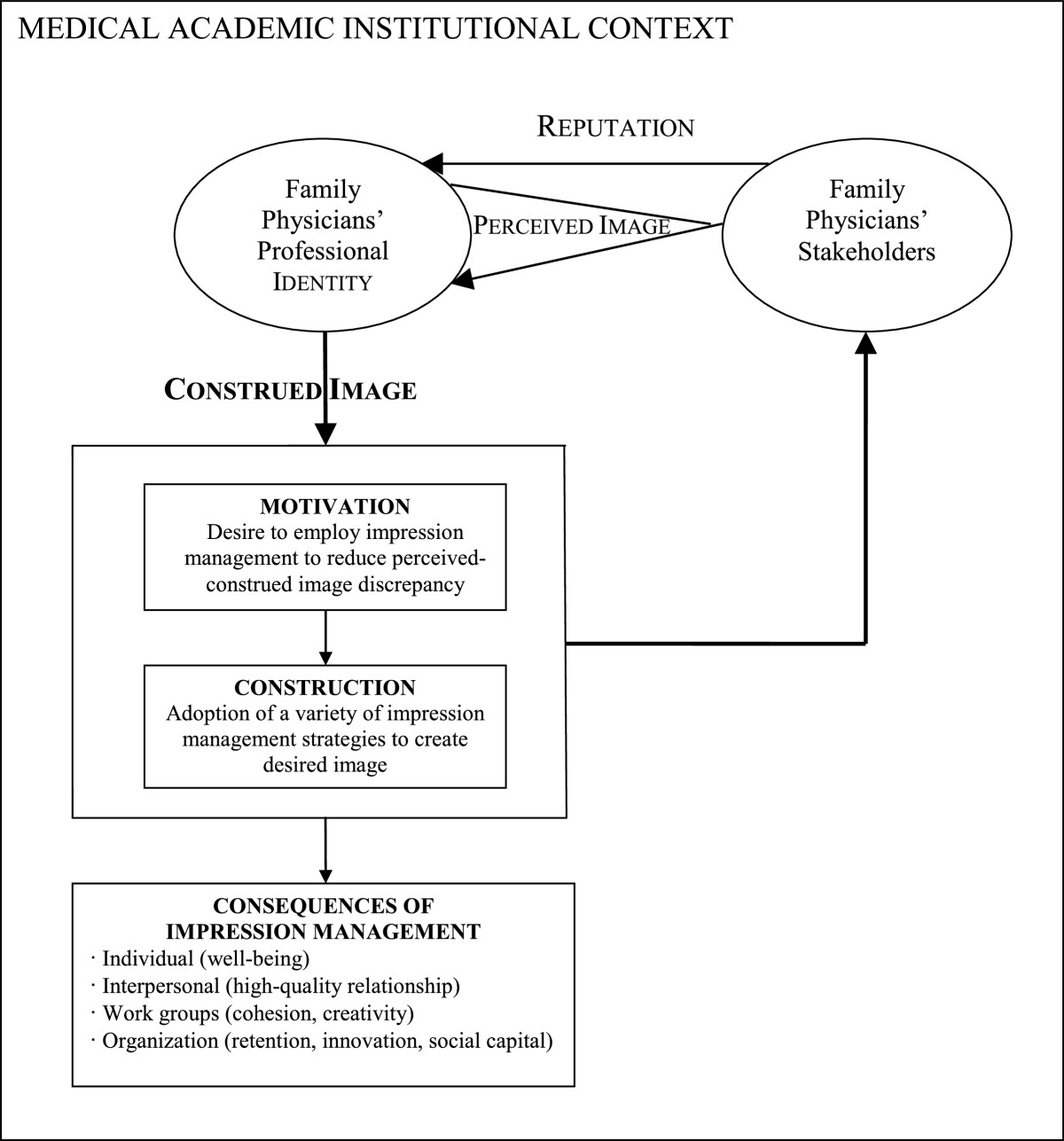
**Impression management in the construction of professional image.**

Until the 1990s, institutional scholars paid great attention to how institutional contexts influence organizations. Following DiMaggio’s remark [83] that institutional theory should integrate ‘agency’ , scholars have become more and more interested in understanding how organizations also shape their institutional contexts. This new trend in organizational institutionalism has been materialized in a number of new topics of interest, such as, among others, the examination of institutional change*,* institutional entrepreneurship and power [84]. Institutional entrepreneurship has been defined as the set of activities displayed by "actors who have interest in particular institutional arrangements and who leverage resources to create new institutions or to transform existing ones" [85]. Institutional entrepreneurship therefore corresponds to the notion of institutional change. Hence, institutional entrepreneurs would therefore be those individual or collective actors to whom institutional change is attributed [86]. Both exogenously-driven institutional change [87] and endogenous source of change [88] have been advocated. In this regard, Suddaby and Viale [89] argue that "the dynamic of professionalization offers an endogenous explanation of institutional change" (p. 425). In other words, these authors emphasize the reciprocal dynamics between processes of institutionalization and processes of professionalization. Based on institutional and profession bodies of literature, these scholars sustain their thesis on two key assertions. First, due to their power attributes, i.e. expertise and legitimacy, professionals are key actors able to mobilize their social capital and skills in driving institutional change. Second, in their quest of securing/enhancing their privileged position, professional projects are closely related to other institutionalization projects, for instance, in universities [90].In sum, in the present study, institutional theory, as well as identity and impression management literature, will help us depict and explain: (1) why and how academic family physicians undertake processes of professional image reconstruction in their institutional contexts, i.e. motivation and construction of impression management strategies; and (2) how this professional project is recursively related to projects of academic institutional change – see also Figure 1.

## Methods/Design

### Research design

We have decided to adopt a *case-based inquiry* as a research strategy. Case study is a very appropriate research design when the researcher asks "why" or "how" research questions and is interested in examining in-depth contemporary phenomena in their naturally-occurring contexts [91]. Our main unit of analysis, i.e. the "case", will be the medical school. Hence, this will be a multiple case study as 4 medical schools from 4 different countries will be involved in the study, namely Canada, France, Ireland, and Spain. It will also be an embedded case study as 4 levels of analysis will be considered: individual family physicians, interpersonal relationships, family physician professional group, and organizations (medical school). Furthermore, as longitudinal research appears crucial for attaining a rich understanding of organizational change [92–94], we will adopt a retrospective-prospective longitudinal design; hence our inquiry is better labelled as a *longitudinal embedded multiple case study*. Such a design appears fully consistent with the topic under investigation, as well as with the current phase of "the maturing of institutional theory" in organization studies, which asks for longitudinal designs and the consideration of multiple levels of analysis [95].

Approval from the McGill Faculty of Medicine Institutional Research Board was obtained in 2013 (A03-E22-13B) while ethics approval from the Research Ethics Committee of the Royal College of Surgeons in Ireland, the *Comité d’éthique du Centre Hospitalier Universitaire de Rennes*, and the *Órgano Evaluador de Proyectos de la Universidad Miguel Hernández* was obtained the present year 2014.

### Case selection

How were our cases selected? A qualitative researcher is not interested in statistical generalizations, but in studying in-depth relatively small samples (even single cases), which are selected *purposively* [96]. The logic and power of a purposive sampling lie in selecting *information-rich cases*; that is cases from which the researcher can intensively learn about the purpose of the investigation [91]. Accordingly, the choice of our four cases responds first to *theoretical concerns* [35]: cases included in the study offer a variety of situational, organizational and societal contexts regarding the topic under investigation. Consequently, to the extent that an international collective case study increases the amount of fieldwork, it will also enable us to elaborate deeper understandings of the complex process of identity construction, and thus, to maximize the possibility of elaborating richer theoretical explanations that may be useful in other contexts. Second, these cases have been selected because of *practical concerns* [35] that mostly relate to our prior research collaboration and the constitution of an international research team for the in-depth examination of processes of family physicians/general practitioners’ identity construction at the international level.

### Data collection

#### Interviews

The main source of data in this study will be both individual and group *interviewing* techniques [97]. Following a purposeful sampling rationale, participants in this investigation will always be *academic family physicians*. This being said, we will contemplate maximum variation sampling [96] with regard to variables such as age/sex, years of academic career and position occupied in the academic hierarchy. We plan to conduct a first round of individual interviews at the beginning of the fieldwork. Inspired by the conceptual framework adopted, the discussion with the participants will focus of the meaning of their professional identity, the awareness of image consistencies/discrepancies, their perceived need and motivations to adopt impression management strategies in order to reduce image discrepancies and enhance professional identity and reputation, and the description and rationale of impression management strategies effectively adopted. We plan to carry out about *60 face-to-face, one-to-one interviews*, i.e. 15 interviews per medical school. Based on our prior research experience in these academic centers, we estimate that this volume of individual interviews will allow us to reach *data saturation*. Interviews will be mostly conducted by members of our research team. Participants will have to sign a written consent form for the interview to be carried out. With their permission, conversations will be tape-recorded, and immediately transcribed and analyzed with the support of the qualitative software package HyperRESEARCH 3.0. Further, we contemplate conducting a series of *focus groups* [98], also with academic family physicians, at least one year after having carried out individual interviews. Our aim with this technique will be to gather participants’ convergent and divergent views with regard to the success or failure of impression management strategies adopted, as well as their intended and unintended consequences at the individual, interpersonal, group, and organizational levels. We plan to conduct a total of *8-12 focus groups*; that is 2-3 focus groups per medical schools. Focus groups will also be tape-recorded, and then transcribed and analyzed with the support of the HyperRESEARCH 3.0 package. They will be conducted by one of the three respective national researchers, with the support of the other two. In order to ensure validity of the data collected, debriefing meetings among the three members of the research team will take place immediately after each focus group.

#### Documentary sources

Documents constitute another important method for collecting data in qualitative research. There are many different types of documents: minutes of meetings, books, manuals, administrative publications, newspapers and magazines, charts, tables, lists, and so on. Documents can also be photos, videotapes, films. And all these documents may already exist, or can be generated throughout the research period. The strengths of a documentary analysis would be the following: on the one hand, documents can help the researcher to elicit nuanced meanings that he or she is trying to understand and, on the other, documents help broaden the understanding of the context surrounding the phenomenon under examination [96, 99]. In the proposed research, archival material will thus enrich our understanding of the academic context within which the family physician’s professional identity is constantly constructed. The fact that all the applicants are professors and/or researchers in the faculties of medicine included in the study will facilitate access to pertinent documents and help identify those truly meaningful for the purpose of the study. Finally, *diary techniques* (i.e. description of any process and event related to the research question observed during fieldwork, summary of main points raised during interviews) complete the portrait of our methods for generating empirical material over the period of inquiry.

### Data analysis

#### Rhetorical analysis

For sake of coherence with the discursive nature of the phenomenon under examination here, we will first and foremost adopt discursive techniques for the analysis of the qualitative material generated. Discourse analysis is considered as the systematic study of texts [100, 101]; in organizational studies, those texts compose and are composed by organizations [101]. There is a great variety of discourse analysis traditions (e.g. discursive psychology, critical discourse analysis, conversation analysis, rhetoric, ethnography of communication, etc.) [35, 100, 102, 103]. In this study, as we are interested in examining how family physicians will try to discursively manage others’ views about the family physician profession, that is how they will use persuasive speech in order to construct an enhance professional image, rhetoric is the discursive approach to adopt. Indeed, impression management will be nothing but rhetorical strategies adopted to promote institutional change [104–107]. Furthermore, "language, particularly rhetoric, plays a central role in how professions reproduce social, cultural and symbolic capital within a field. Language is a crucial weapon in this process and professionals are skilled rhetoricians" [89]. Rhetorical analysis, which implies the description and interpretation of how effectively a text persuades and convinces an intended audience [108, 109] will be applied to both texts from interviews and documents gathered. Our organizational rhetoric analysis, largely influenced by critical theory [108], will be performed by each national group, under the coordination of the principal investigator.

#### Within-case and cross-case analysis

Due to the fact that our study involves more than one case, as well as different levels of analysis and different type of data, we will undertake two major analytical steps, i.e. within- and cross-case analysis stages [110]. Within-case analysis will let us describe, understand and explain how family physicians operating in a particular institutional context construct their professional image, and with what immediate effects. Then, cross-case analysis will allow us recognize and explain regularities and variations among the four international medical schools involved in the investigation.

## Discussion

The results of our study will potentially have important implications for the practice of family medicine, medical education, and health and educational policies. The processes by which medical students’ choose a residency program are complex, relying on a multiplicity of arguments that can be located at different levels, from personal to societal dimensions. Family medicine stakeholders (professionals, patients, policy decision-makers) of different health care systems have mostly focused on improving the working conditions of family physicians/general practitioners once they graduate. This being said, we argue that several interrelated initiatives could be undertaken in medical schools to help generate *new* institutional academic discourses that contribute to the formation of an enhanced family medicine professional identity. As our prior investigation has highlighted, unanimous agreement exists among medical students of several countries with regard to the lack of prestige of family medicine, reinforced through undergraduate training in academic contexts that clearly associate medical expertise and excellence with specialized medical knowledge. Such a low reputation of the profession is also reinforced by health care system and societal contexts where specialized medical knowledge is most valued, and may prevent even students who may initially be interested in this professional practice from finally choosing this path. To further reverse this trend and, therefore, support students’ identification with this profession, more attention should be paid to family physicians’ professional identity formation processes in medical schools; further research exploring such processes should thus be conducted. This is the commitment of our international research team, which in the present investigation aims to examine how academic family physicians reconstruct their professional image. As noted by Cooke and collaborators as closing remark of their 2010 book [55], "preparing physicians who have a firm professional identity, who continuously seek excellence through inquiry, and who are engaged as members of a moral community will ensure the highest-quality care for patients". Indeed, the results of our investigation will thus accomplish what Rist [111] qualifies as the "enlightenment function" of policy research: "Viewing policy research as serving an enlightenment function suggests that policy researchers work with policy makers and their staffs over time to create a contextual understanding about an issue, build linkages that will exist over time, and strive constantly to educate about new developments and research findings in the area" (p. 1003).

While we are deeply interested in supporting the practice of family medicine and education in the discipline, we also intend to further contribute to knowledge generation about professional identity and organizations. In this regard, the research proposed here is original and enhances the literature in organizational studies and health services and policy research in a number of ways. First, as pointed out in the literature review of this protocol, not only are theoretical contributions to professional image construct scant, but empirical research on family physicians professional image construct is inexistent. Second, the investigation is even more challenging and original as we propose to undertake an international research investigation that examines such processes in very different academic, institutional and national contexts. Third, the investigation is also innovative because it adopts a process approach and a longitudinal design. As noted by the organizers of the Symposium ‘Doing Longitudinal Studies of Health Care Change: Studying Health Care Change’ at the last Academy of Management Annual meeting: "In-depth longitudinal case studies of health care change are needed to better understand how these processes occur and how they might be more successfully managed" [112]. In the end, the present investigation is also innovative in regard to the theoretical perspective adopted, i.e. institutional theory. More specifically, the originality of this study is related not only to the use of one of the most compelling theories in current organizational and management literature but also to its combination with identity and image corpus of knowledge, as well as our focus on the intertwinement between professions and organizations. As noted by Greenwood and collaborators [81]: "In its own neglect of the more micro-dynamics of sensemaking, institutional theory has relinquished the opportunity to develop a richer theory of the intersubjective processes of perception, interpretation and interaction that establish the core of a micro-level understanding of institutionalization" (p.30).

## Authors’ original submitted files for images

Below are the links to the authors’ original submitted files for images. Authors’ original file for figure 1
