# Supplementary figures and images for: Family physicians’ professional identity formation: a study protocol to explore impression management processes in institutional academic contexts
Source: BMC Med Educ. 2014 Sep 6;14:184. doi: 10.1186/1472-6920-14-184 (PMC4167519; doi:10.1186/1472-6920-14-184)

MEDICAL ACADEMIC INSTITUTIONAL CONTEXT

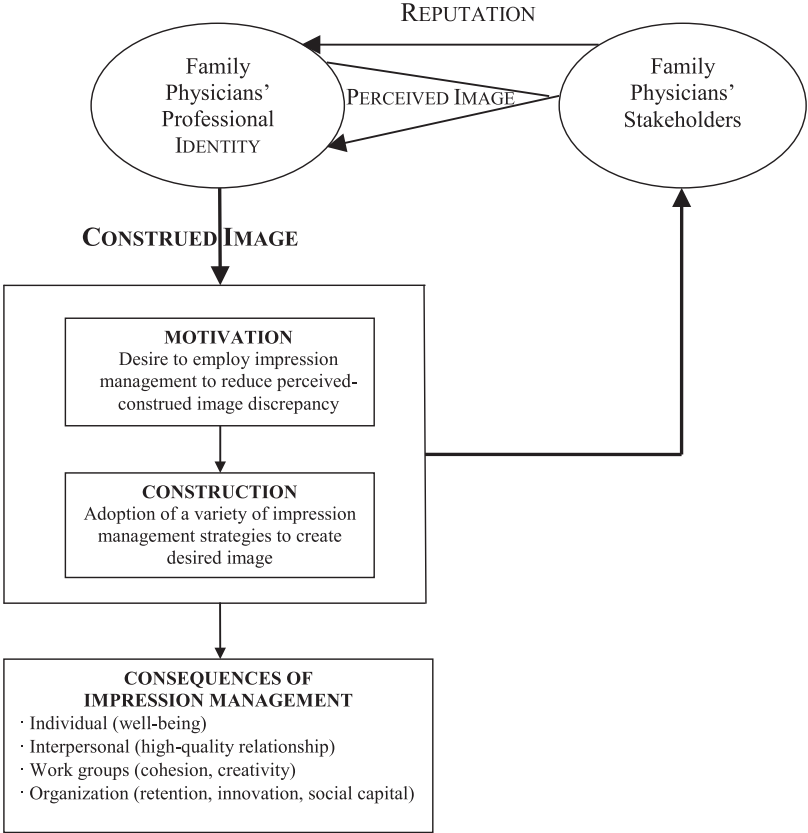

Supplement: Supplementary file 1 — Authors’ original file for figure 1 [file 12909_2014_1013_MOESM1_ESM.pdf]
